# Unravelling the Effectiveness of Anthelmintic Treatments on Equine Strongyles on Irish Farms

**DOI:** 10.3390/ani14131958

**Published:** 2024-07-02

**Authors:** Nagwa Elghryani, Amanda Lawlor, Trish McOwan, Theo de Waal

**Affiliations:** 1 School of Veterinary Medicine, University College Dublin, D04 W6F6 Dublin, Ireland; amanda.lawlor@ucd.ie (A.L.); theo.dewaal@ucd.ie (T.d.W.); 2 Telenostic Limited, R95 WN20 Kilkenny, Ireland; trish.mcowan@telenostic.com; 3 Department of Biology, Faculty of Arts and Sciences-Gamines, University of Benghazi, Benghazi 1308, Libya

**Keywords:** FECR, equine strongyle, anthelmintic resistance, Irish equine farms, cyathostomin

## Abstract

**Simple Summary:**

The global prevalence and diversity of equine strongyles pose a significant challenge in the equine industry, exacerbated by the widespread development of anthelmintic resistance within this nematode group. In the specific context of Ireland, there is a lack of conclusive information regarding the efficacies of various anthelmintic classes employed for helminth parasite control in equines. This study aims to assess the effectiveness of commonly used anthelmintics through a fecal egg count reduction test. Individual egg counts were measured on day 0 (pre-treatment) and day 14 (post-treatment) using the mini-FLOTAC technique. Pivotal findings of this research were the widespread benzimidazole resistance and the evidence of resistance to ivermectin and moxidectin.

**Abstract:**

Over the preceding decades, the widespread dependence on anthelmintic drugs for managing nematodes in grazing equids has given rise to resistance against commonly used anthelmintics in various countries. This study explores the prevalence of anthelmintic resistance across 44 horse farms in Ireland. Anthelmintic efficacy was evaluated through fecal egg count reduction (FECR) tests employing the mini-FLOTAC technique. Resistance to benzimidazoles was identified in 12 out of 14 farms (FECR range: 0.00% to 86.2%). Ivermectin resistance was observed on two farms, one with an FECR of 80.70% and another with an FECR of 96.10% (lower 95% high probability density interval (HPD) <90%, 11.70%). On the remaining six farms, the reduction with ivermectin still exceeded 95%. The reduced efficacy of moxidectin was noted on two farms (FECR = 86.90% and 93.50%) and on a third farm with an FECR of 99.50 and a lower HPD interval < 90% at 24.00%. In summary, these findings emphasize the urgent need for alternative strategies in equine strongyle control that reduce reliance on anthelmintics and prioritize effective management practices on Irish equine farms to hinder the impending development of drug-resistant parasite populations.

## 1. Introduction

The high prevalence and diversity of cyathostomins (small strongyles) continue to be a significant threat to the health of horses, causing serious economic losses, especially since the development of widespread anthelmintic resistance (AR) in this group of nematodes [1,2,3,4,5,6]. These infections have been described as being responsible for decreasing weight gain and causing colic, diarrhoea, anemia, and even mortality in horses [5].

The first report of cyathostomin resistance to benzimidazole (BZ) was recorded in 1966 by Drudge and Lyons [7]; since then, it has been extensively reported in many countries [8,9,10]. With increasing levels of resistance to pyrantel also being reported [8,11,12], macrocyclic lactones (ML), such as ivermectin (IVM) and moxidectin (MOX), have now become the most commonly used drug class to control horse helminths [13,14,15,16]. However, the trend in cyathostomin control is towards the application of anthelmintics to all animals in all age groups in the field without attempting to test efficacy or perform faecal examinations. Several studies over the last two decades have reported a shortened egg reappearance period (ERP) following treatment with IVM and MOX, which have been considered early indicators of changes in drug efficacy [17,18,19,20]. Recent meta-analyses have also supported this consistency across many countries [20]. This analysis conducted a comprehensive examination of ERP data concerning three primary anthelmintic classes and included 54 studies spanning from 1972 to 2022, which were identified and met the inclusion criteria for this systematic review. Notably, up until the beginning of 2022, a consensus on the definition of ERP was lacking, resulting in the identification of eight distinct definitions in literature, which posed significant challenges for comparative analysis between studies. Factors contributing to the shortening of ERP, such as previous anthelmintic usage and climatic conditions, were not elucidated. Comparative ERP definitions revealed MOX and IVM ERPs of 35 and 28 days, respectively. However, the underlying reasons behind these reduced ERPs remain ambiguous, whether stemming from AR development or other biological factors, such as the host, cyathostomin species, or environmental influences. 

Not surprisingly, IVM and MOX resistances in cyathostomins have also recently been identified [2,11,17,18,19,20,21]. However, many studies have shown that >80% of the worm burden is harbored by around 20% of the horses within a population [16,22,23,24]. Therefore, the identification of high egg-shedders and the use of anthelmintics with targeted treatment programs would potentially slow down the development of AR [25]. It is therefore advisable (i) to determine the effectiveness of the anthelmintics in use and (ii) to only use anthelmintics with the highest efficacy. Another factor that contributes to the appearance of the resistance is the importation of horses from regions where there is already resistance. One example of this occurred in 2019, when a cohort of thoroughbred yearlings was imported from Ireland to the USA. Following the administration of IVM to 110 yearlings from different groups (USA-bred yearlings and imported yearlings) in February 2020, fecal egg count reduction (FECR) analysis revealed a 100% reduction in USA-bred yearlings. Conversely, imported yearlings (*n* = 59) exhibited FECR rates of 93.5%, 70.5%, and 74.5% in three distinct groups. Subsequently, the two former groups of imported yearlings underwent further retreatment with IVM, resulting in lower FECRs of 33.8% and 23.5%, respectively. These horses were then subjected to treatment with either MOX or a triple combination of MOX, oxibendazole, and pyrantel pamoate. MOX-treated groups exhibited FECRs of 90.2%, 57.3%, and 50.0%, while the triple combination achieved a 100% FECR in all treated groups. To confirm ML resistance, a reassessment of IVM efficacy was conducted in June 2020. Imported groups displayed FECRs of 99.8%, 87.7%, and 62.0%, indicating persistent resistance. In contrast, USA-bred yearlings maintained an FECR within the 99–100% range. This study unequivocally illustrates the presence of ML resistance in cyathostomins imported from Ireland and underscores the potential for the global dissemination of ML-resistant parasite isolates through the movement of horses [26].

According to the World Association for the Advancement of Veterinary Parasitology (WAAVP), the FECR test is the most appropriate for determining drug efficacy, where pre-treatment egg excretion is compared with egg excretion 14 days post-treatment to calculate the percentage efficacy [27,28,29]. Recently, research has addressed the question of how best to analyze data from an FECRT using Bayesian models [30,31]. Drugs are considered to be efficacious when the FECR is >95% for BZ or >96% in the case of IVM and MOX [29]. 

Studies in the USA have shown that a 4-week ERP after IVM treatment is associated with resistance in the luminal L4 (fourth-stage larvae) of cyathostomins, where the IVM efficacy was <80% against the luminal L4 stage. The situation of MOX resistance is less clear, as despite a shortened ERP after treatment, the efficacy against luminal L4 stages ranged from 96% to >99% [32,33,34,35,36]. A recent study in 2023 [19] conducted on horse farms in the USA highlighted that two weeks after treatment, both MOX and IVM exhibited high effectiveness against adult worms (99.9% and 99.7%, respectively), but their respective efficacies against luminal L4 were 84.3% and 69.7%. Five weeks post-treatment, the adulticidal efficacy was 88.3% for MOX and 57.6% for IVM, while neither drug affected luminal L4. MOX reduced early L3 counts by 18.1% and 8.0% at 2 or 5 weeks, respectively, while efficacies against late L3 (third-stage larvae) and mucosal L4 were 60.4% and 21.2% at the same intervals [19].

As far as the authors are aware, no studies have been published to date in Ireland on the efficacy of anthelmintics against cyathostomins currently available to, and most frequently used by, Irish horse owners. This study aims to determine the efficacies of the commonly used anthelmintic drugs, using the FECR test, in participating Irish equine farms.

## 2. Materials and Methods

### 2.1. Study Population

#### 2.1.1. Farm Selection

Between January 2015 and October 2021, a total of 575 horse from 44 horse farms took part in the investigation. Most of them also participated in the questionnaire survey in 2014 [13]. Each participating farm had a minimum of eight permanent resident horses. The owners consented to repeated samplings, as well as the administration of the anthelmintic drugs. The owners followed their routine treatment schedules and treated the animals with their drug of choice, which was either BZ or a ML (IVM or MOX), at the recommended dose rates of 7.5, 0.2, and 0.4 mg/kg of body weight, respectively. On a few farms (*n* = 4), the horses were treated with a five-day dose of BZ. 

#### 2.1.2. Sample Collection

An addressed envelope including instructions for sample collection, fecal containers, and a submission form to complete was sent to each participant. This form was designed to provide information on the animal (name, age, and gender) and anthelmintic usage (date of last dewormer and last-used dewormer). Each animal owner or farm manager was asked to collect at least two fresh fecal boluses on the day prior to deworming (day 0). The following day, the anthelmintic drug was administered by the owners or managers. The fecal samples were again taken 14 days post-treatment. Fecal material was collected from freshly voided feces by the horse owners. The samples were sent to the laboratory immediately after collection and stored at 4 °C upon arrival. All samples were processed as soon as possible but always within four days.

### 2.2. Faecal Egg Count Reduction Test

All available horses or up to 43 random animals examined on Day 0 were included in the study. Individual egg counts were determined from each animal on Day 0 (pre-treatment) and Day 14 after treatment using a mini-FLOTAC technique with a minimum detection limit of 5 or 7.5 epg [37], depending on whether 5 g of fecal sample with 45 mL of flotation solution or 3 g of fecal sample with 42 mL of flotation solution was used. Feces (3 g or 5 g) was added to a graduated measuring cylinder that contained, respectively, either 42 or 45 mL of saturated sodium chloride solution (NaCI, specific gravity 1.2) and was well-homogenized using a spatula. The suspension was then filtered through a tea strainer and collected in a beaker. The prepared suspension was loaded into each of the chambers of the mini-FLOTAC (University of Naples Federico II, Naples, Italy). After 10 min, strongyle eggs were counted in both chambers using a microscope (Optika Microscope model B-800BF, Ponteranica, Italy) at 100× magnification 

### 2.3. Data Analysis

The FECR was calculated for each group of animals following the WAAVP guidelines [27] and using the online eggCounts package (eggCounts-2.3-2 on R version 4.4.1) developed by Wang et al. [30,31]. As there are no standard guidelines for appropriate cut-off limits for the FECR test in horses, this study followed previously published threshold recommendations, where the efficacy was set at an arithmetic mean FECR of >95% and/or a lower 95% confidence interval >90% [27].

## 3. Results

A total of 575 horses from 44 farms participated in the FECR study. From 14 farms, 92 horses were tested for a single dose of BZ efficacy, and 26 horses from 4 farms were tested for the efficacy of BZ using a five-day dose. The efficacies of IVM and MOX were tested on 101 and 356 horses across 8 and 18 farms, respectively. Before the start of this study, the horses on F19 were treated with pyrantel pamoate (PYR); on F07, F09, and F23, horses were treated with BZ, whereas on the rest of the farms, the horses were treated with ML.

FECR was conducted on 14 farms using a single dose of BZ. BZ resistance was observed in 85.7% (*n* = 12) of these farms, with FECR ranging from −100% to 86% (Table 1). On the four farms that followed a five-day BZ treatment, resistance was identified in two of the farms (Table 1).

The efficacy of IVM remained above the 95% cut-off on 75% of the farms (6/8). However, Ivermectin resistance was observed on two farms: F25, where the FECR = 80.70%, and F24, where the FECR was 96.10%, with the lower 95% high probability density interval < 90% (11.70%) (Table 2).

MOX was effective in 83.33% (*n* = 18) of the farms, with an FECR ranging from 99.80% to 100% (Table 2). However, resistance to MOX was detected on three farms (F37, F43, and F44) (Table 2).

## 4. Discussion

Gastrointestinal parasitism stands as a significant economic burden within the global equine industry. Despite its prominence, research linking equine health to parasite infections in Ireland remain scarce. Among the few studies conducted, strongyle infection emerges as the predominant nematode concern. In a comprehensive examination of 2700 horses, it was shown that 52.4% of them excreted strongyle eggs. Intriguingly, only 32% of these horses were found to be responsible for excreting 80% of the total detected strongyle eggs. This highlights the disproportionate impact a minority of infected horses can have on parasite transmission within the equine population [22]. Cyathostomins is recognized for its substantial epidemiological significance, attributed to its widespread resistance to anthelmintics. Moreover, no information is available on the anthelmintic efficacy statuses of various anthelmintic drug classes used on Irish horse farms. The goal of this study was to assess the anthelmintic efficacy against equine strongyles in Ireland. Resistance to BZ is common in strongyle populations across the world [17,20,38,39,40,41,42,43,44,45,46,47,48,49,50,51,52,53,54,55]. In our study, a lowered efficacy of a single dose of BZ, as determined by FECR test, was detected in 12 of the 14 farms investigated, where the efficacy ranged between 41.0% and 86.2%, highlighting the widespread reduced BZ efficacy against equine strongyles in Ireland. The results follow a similar trend in studies in Europe. An extensive European survey conducted by Traversa in 2009 [8] reported that the single-dose use of fenbendazole (FBZ) was ineffective in 82.4% of the 17 yards examined in the UK, with reduced efficacy observed in almost all German yards (84.6%). In Italy, the efficacy of FBZ was found to be reduced in approximately one-third of the yards (38%). Another study in the UK reported that the efficacy of BZ ranged from 0.4% to 41% in a study involving 30 horses from three thoroughbred studs [17]. In Sweden, Linda et al. in 2007 [40] reported resistance to BZ in 76.9% of the yards, with a further 23.1% of yards having suspected resistance cases. A recent report from Lithuania [10] revealed that although all anthelmintics intended for use in horses are available by prescription only, there is a high level of resistance observed. The BZ showed reduced efficacy, with the FECR ranging from 71.1% to 79.0% in half of the stables. Suspected reduced efficacy was also noted in one horse stable, with the FECR at 93.6%. In 58 studies [9], the efficacy of BZ was assessed in 31 countries and six continents, and resistance was reported in every single one of them. A study reported resistance to FBZ and oxibendazole, with efficacies of 6% and 20.83%, respectively. This resistance was observed in 65 and 48 horses, respectively, from different farms located in the mid-Atlantic region of the USA [43]. Resistance to BZ with FECRs of 41.1–62.3% and 53.3–66%, respectively, was reported in Algeria [44]. Considering all the aforementioned data, high prevalences of strongyles resistant to BZ have been reported globally since 2000. This is of great concern, as a recent survey in 2019 [13] indicated that BZ is still widely used by Irish horse owners (53%). Among the farms tested in 2015 for the efficacy of BZ, two farms (F13 and F14) treated all animals monthly, while the others treated all animals more than four times a year. Additionally, farms tested in 2020 (F02, F03, F04, F06, and F09) administered anthelmintics more than three times a year. These farms exhibited indiscriminate usage of anthelmintics with improper dosage, which stands out as a crucial factor influencing the development of resistance alleles [45]. The available evidence suggests that anthelmintic resistance persists over numerous years and across multiple parasite generations once it emerges within a parasite population. Furthermore, once anthelmintic resistance is established in cyathostomins against a specific anthelmintic class, there is minimal expectation for a return to susceptibility [9]. A study published by Lyons identified BZ resistance in a cyathostomin population untreated for 22 years. Upon re-evaluation, BZ resistance persisted. In a subsequent study, ponies harboring BZ-resistant cyathostomins were treated bi-monthly with PYR for 8 years. Post-assessment showed persistent BZ resistance and the emergence of PYR resistance [9]. The widespread BZ resistance worldwide has resulted in the majority of current deworming programs relying on ML to control strongyle infections [13,14,15,16]. The frequent and indiscriminate use of ML will inevitably lead to the development of resistance. There is evidence of a shift in MOX and IVM efficacies, either through shortened ERP or low FECR being reported in several studies [2,11,17,18,19,20,21,46,47,48,49,50,51,52]. In a subsequent report by Nielsen in 2022 [9], MLs were assessed in 57 studies conducted since 2000, revealing evidence of resistance to this class in 13 of them (23%). This study confirmed the presence of IVM resistance on two farms (F25: FECR; 80.7%) and F24, where the FECR was 96.10% but with the lower 95% HPD interval < 90% (11.70%). However, on the other six farms, the IVM reduction was still >95%. Moreover, MOX demonstrated high efficacy rates, with the FECR ranging between 99.5–100% on 18 farms, consistent with findings in other studies [52,53,54]. However, two farms (F43; *n* = 21 and F44; *n* = 8) exhibited a reduced efficacy of MOX, with FECR rates below 95% (86.9% and 93.5%, respectively), and on another farm, F37, the FECR was 99.50%; however, the lower HPD interval was <90% (24.00). This is, however, not the first report of IVM and MOX resistances in Irish horse farms, as both IVM and MOX resistances have previously been reported by Nielsen et al. [26] in yearlings imported into the USA from Ireland. The treatment history of the farms in this study suggests that over-treatment and underdosing of horses are the main factors contributing to the emergence of resistance. The reduced efficacy of ML in this study may also be attributed to specific cyathostomin species and poor management systems. 

Reduced efficacy of ML against horse strongyles has been reported worldwide. In the UK, several studies have indicated resistance to ML [8,17]. The most recent study in the UK confirmed strongyle resistance to ML and PYR from one of four studs sampled in 2021, with FECR ranging from 36.4% to 78.6% (95% CI:15.7–86.3) after IVM treatments and with FECR being 72.6% (95% CI: 50.8–85.2) after MOX treatment and 80.8% (CI: 61.9–90.0) after treatment with PYR. In 2022, only IVM was tested, once again revealing the presence of resistant cyathostomins infecting young stock. However, another stud tested in the same year showed a four-week ERP with an FECR of 98% (CI: 95.4–99.2%), and MOX was effective in the other three studs, exhibiting a short ERP of six weeks [56]. A French study identified the reduced efficacy of IVM in one animal on one farm and of MOX in one animal on another farm [54]. The situation is even getting worse, with cases of multidrug resistance against more than one chemical class being documented. For example, Traverse et al. [8] tested FBZ, PYR, and IVM in horse studs across Europe and found that among the sampled farms, 20% (*n* = 50) in Italy, 29.4% (*n* = 17) in the UK, and 23.1% (*n* = 13) in Germany exhibited multiple resistance. The combination of FBZ and IVM was consistently observed in cases of multiple resistance on one of the Italian and UK farms, while another UK farm showed FBZ and PYR resistances and also suspected resistance to IVM because the FECR was >90%, and the lower 95% confidence limit was < 90%. More worrying was the detection of triple resistance on one UK farm, where resistances were detected against FBZ, PYR, and IVM. Another recent French study [53] also reported a triple resistance to FBZ, PYR, and IVM in three groups of yearlings. In 2020, the reduced efficacy of ML in equine strongyles in the USA was reported [26] in 110 yearlings; it was interesting that there was documented resistance to ML among the 59 imported yearlings, while the US-born ones displayed full efficacy on the FECRT. Subsequent research revealed IVM resistance in a group of US-born yearlings raised on a different farm in the same area, indicating domestic ML resistance in the USA equine population [9]. Over the past decade, compelling evidence of ML resistance among cyathostomins has also surfaced in Brazil. Six studies, comprising nearly half of all investigations on ML resistance, have documented diminished efficacies across various age groups within this anthelmintic class. [9,21,46,48]. Furthermore, MOX resistance was recently demonstrated in groups of weanlings and yearlings in Australia [52]. 

Instances of a reduction in strongyle ERP after MOX treatment with a 100% effective FECR have been reported over the past several decades on horse farms in the UK [8,17,18,56,57], Europe [58,59], the USA [50,51], and Australia [52]. Taken together, these recent data suggest that a long-awaited breakthrough of ML resistance in equine strongyles has finally happened and that many more reports can be expected in the coming years. The decrease in ERP post-treatment could signify early indications of the diminished efficacies of IVM and MOX against immature small strongyles, potentially resulting in the incomplete elimination of cyathostomins from the intestinal lumen or a shortened maturation period for the parasite [19,32,33,34,35,36]. An alternative explanation may involve the selective treatment of certain species within the diverse Cyathostominae subfamily, comprising over 50 equid species, each with varying pre-patent periods, where a single horse can harbor multiple species [3,19,59]. In Irish studies conducted by Byrne et al. (2023) [60] and Kinsella (2002) [61], the most prevalent species globally were also highly represented. Byrne et al. [60] identified *Coronocyclus* (*Cor.*) *coronatus* and *Cylicocyclus* (*Cyc*.) *nassatus* as the most prevalent, occurring in 64% of the sampled horses, while Kinsella [61] recorded four species with 100% prevalence, namely *Cyc. nassatus, Cyathostomum* (*Cya.*) *catinatum, Cyc. calicatus*, and *Cyc. longibursatus*. Similarly, Bellaw and Nielsen [3] reported the highest prevalences for *Cyc. nassatus*, *Cya. catinatum*, and *Cyc. longibursatus.* Recent studies have highlighted the contribution of the *Cylicocyclus* species to a shortened ERP [19,59]. Investigations on cyathostomin populations from ML-resistant horses in 2022 demonstrated that *Cyc. nassatus,* among other *Cylicocyclus* species, played a significant role in ERP reduction. Subsequent studies by Nielsen in 2023 [26] underscored that shortened ERP led to a notable decline in the anthelmintic efficacies of IVM and MOX, with treatment intensity exacerbating this effect. In an Irish study by Elghryani [62], efforts were made to identify surviving cyathostomin species under different drug concentrations in vitro and larval migration inhibition assays (LMIA), revealing the consistent prevalence patterns across various regions, with *Cyc. nassatus* being the most common. These findings align with previous studies worldwide, suggesting an association between *Cylicocyclus* species predominance in horses and shortened ERPs following IVM or MOX treatment. Furthermore, the same *Cylicocyclus* species were identified in both IVM and MOX LMIAs, indicating their prevalence in ML-resistant populations.

Several factors have been identified that contribute to the emergence of AR, with frequent anthelmintic treatment being a prominent factor in the selection of resistant helminth populations. Inaccurately calculated dosages and errors in weight estimation, especially through the prevalent method of visual weight estimation, may contribute significantly to this outcome [39]. Not maintaining a nematode population in refugia is also a contributing factor [9,39]. Over two decades, several studies in Ireland on horse parasite control programs have shed light on the concerning trend of overreliance on anthelmintic control programs, especially ML, with little or no diagnostic monitoring of parasite presence or treatment efficacy [13,63,64]. Despite consistent advice on the best practice of parasite control, horse owners are reluctant to take this onboard. In an effort to try and explore the reasons behind that, Walshe et al. [64] recently conducted a study exploring the factors influencing parasite control practices among Irish equine thoroughbred breeders and identifying barriers to the adoption of sustainable strategies on their farms. Guided by principles from behavioral science, their research sought to understand and alter behaviors beyond merely disseminating information or resources. Employing qualitative research methods and inductive thematic analysis, the study prioritized the perspectives of equine owners over those of parasitology experts or advisors. This approach ensured that interventions and recommendations are customized to meet the specific needs of end-users. Furthermore, the research underscored the importance of active engagement from end-users, as legislative changes alone are unlikely to catalyze improvements in equine parasite management. Traditional pasture maintenance practices overshadow biosecurity measures, and participation in parasitology diagnostics remains limited. Equine veterinarians typically are not involved in providing parasitology guidance, while concerns regarding anthelmintic resistance are perceived as broader industry-wide challenges rather than immediate concerns at the individual farm level. Following the completion of our study, the guidelines for evaluating anthelmintic resistance in farm animals have been updated. The new guidelines [29] aim to improve AR detection by optimizing experimental design and statistical analysis tools, enabling more accurate interpretations of FECRT data and providing correct classifications of resistance status and drug efficacy. The newer protocols are expected to detect even small changes in drug efficacy.

## 5. Conclusions

The findings of this study underscore the urgent need for alternative approaches to equine strongyle control that are less reliant on anthelmintic use and more focused on effective management practices aimed at reducing environmental contamination and parasite challenges. 

In light of these findings, it is evident that strategies to combat anthelmintic resistance in horse populations must prioritize the implementation of sustainable management practices. These may include improved pasture management, strategic grazing protocols, regular manure removal, and targeted deworming based on evidence-based guidelines rather than a fixed treatment schedule. By adopting a holistic approach that integrates both anthelmintic use and management practices, horse owners and managers can work towards mitigating the spread of AR while safeguarding horse health and welfare in the long term. 

If horse farms fail to adjust their approach to utilizing the remaining effective drugs, we may soon face a situation where these parasites cannot be effectively controlled. This issue poses a significant threat to horse farms, potentially impacting profitability and forcing more farms out of business. To address this challenge, horse owners/farmers must shift their perspective on parasite control and make fundamental changes to how they care for their animals.

## Figures and Tables

**Table 1:** The fecal egg count reduction (FECR) data and the high probability density interval (HPD) for benzimidazole (BZ) treatment. The number of horses and mean strongyle fecal egg counts (FEC) expressed in egg per gram (epg) are shown for day 0 and day 14, along with the FECR percentage.

Farm Code	Number of Animals	Day 0 FEC (epg) Mean	Day 14 FEC (epg) Mean	FECR%	HPD Interval
Single dose (7.5 mg/kg)					
F01	4	1417.20	0.40	100.00	99.7, 100
F02	4	1426.87	476.01	64.10	60.5, 66.5
F03	8	1440.68	1436.56	0.00	0.00, 0.005
F04	5	1049.90	517.80	49.60	21.0, 68.5
F05	7	1038.54	549.86	46.60	40.6, 51.7
F06	4	1583.45	705.20	55.80	50.5, 60.3
F07	5	415.41	186.62	54.90	45.0, 63.5
F08	17	1142.8	612.94	61.00	28.0, 87.0
F09	3	1487.81	796.97	48.60	42.6, 54.7
F10	2	424.22	1.03	99.80	97.5, 100
F11	2	677.19	392.51	41.60	27.2, 54.0
F12	1	677.27	277.71	56.10	16.0, 79.1
F13	7	207.69	30.01	86.20	81.6, 90.1
F14	23	371.95	144.36	61.60	58.0, 64.8
Five-day dose (7.5 mg/kg/day for five days)					
F15	5	828.70	36.50	95.40	93.6, 97.1
F16	8	933.40	0.23	100.00	99.7, 100
F17	5	415.41	186.62	54.90	45.0, 63.5
F18	8	381.34	92.40	74.60	69.8, 78.9

**Table 2:** The fecal egg count reduction (FECR) data and the high probability density interval (HPD) for ivermectin (IVM) and moxidectin (MOX) on horse farms. Number of horses and mean strongyle fecal egg counts (FEC) expressed in egg per gram (epg) are shown for day 0 and day 14, along with the FECR percentage.

Farm Code	Number of Horses	Day 0 FEC (epg) Mean	Day 14 FEC (epg) Mean	FECR%	HPD Interval
IVM (0.2 mg/kg)					
F19	3	828.70	36.40	95.40	93.6, 97.1
F20	3	933.40	0.20	100.00	99.7, 100
F21	21	833.80	100.00	99.90	99.2, 100
F22	3	889.80	0.50	99.90	99.3, 100
F23	25	418.50	0.00	100.00	99.9, 100
F24	6	10.30	0.92	96.10	11.7, 100
F25	20	483.22	89.22	80.70	77.3, 84.1
F26	20	505.80	7.86	98.40	96.9, 99.3
MOX (0.4 mg/kg)					
F27	8	624.40	0.30	100.00	99.6, 100
F28	23	581.42	0.04	100.00	99.9, 100
F29	14	165.61	0.08	100.00	99.3, 100
F30	12	1092.60	0.08	100.00	99.9, 100
F31	12	332.58	0.10	100.00	99.6, 100
F32	21	189.48	0.05	100.00	99.6, 100
F33	29	271.90	0.04	100.00	99.8, 100
F34	30	519.60	0.00	100.00	99.9, 100
F35	20	110.29	0.29	99.80	98.6, 100
F36	19	304.95	0.06	100.00	99.7, 100
F37	6	157.50	0.00	99.50	24.0, 100
F38	38	224.37	0.04	100.00	99.7, 100
F39	43	520.55	0.14	100.00	99.9, 100
F40	12	717.73	0.08	100.00	99.8, 100
F41	20	502.56	0.05	99.90	99.8, 100
F42	20	204.00	0.05	100.00	99.7, 100
F43	21	84.82	11.31	86.90	73.8, 94.8
F44	8	24.72	2.33	93.50	31.6, 99.4

## Data Availability

The data presented in this study are available upon request from the corresponding author. The data are not publicly available.
